# Chronic Nipple Sinus Secondary to Demodex folliculorum Infestation: A Case Report

**DOI:** 10.7759/cureus.85889

**Published:** 2025-06-12

**Authors:** Suhair Al Saad, Hamdi Al Shenawi, Kameela S Majed, Khalid Al Sindi

**Affiliations:** 1 Surgery, Arabian Gulf University, Manama, BHR; 2 General Surgery - Breast Oncoplastic and Reconstructive Surgery, Ivan Mikhailovich Sechenov Medical University, Moscow, RUS; 3 Pathology, Blood Bank and Laboratory Medicine, King Hamad University Hospital, Al Sayh, BHR

**Keywords:** breast, demodex folliculorum, demodicosis, mastitis, nipple-areola sinus

## Abstract

*Demodex folliculorum* mites are common commensals of human skin. They typically inhabit pilosebaceous units. While often asymptomatic, infestation can lead to demodicosis. Involvement of the nipple-areolar complex is rare and can mimic other benign or malignant conditions.

Herein, we report a case of a 30-year-old, healthy, single woman who initially presented with symptoms suggestive of a simple breast cyst. Eight months later, following a severe COVID-19 infection, she developed a discharging periareolar ulcer with associated nipple retraction. Surgical exploration revealed a sinus tract connecting the nipple to the ulcer. Histopathology of the excised tissue unexpectedly demonstrated heavy infestation of pilosebaceous units with *D. folliculorum* mites within chronic granulation tissue. Following surgery, the patient was treated with oral metronidazole and topical agents (tea tree oil wash and sulfur soap).

## Introduction

*Demodex folliculorum* is a microscopic mite - a common ectoparasite residing within the pilosebaceous follicles of human skin, particularly on the face and eyelids. Its prevalence increases significantly with age, reportedly found in 25% of 20-year-olds, 30% of 50-year-olds, and potentially 100% of individuals over 90 [1]. While typically considered a harmless commensal, an overgrowth or altered host immune response can lead to various cutaneous manifestations, collectively known as demodicosis [1-3]. Immunosuppression (e.g., secondary to diabetes, chemotherapy, or certain medications like topical steroids) can predispose individuals to higher mite populations and subsequent inflammation [3,4]. Clinical presentations can range from subtle erythema, scaling, and pruritus (pityriasis folliculorum) to more pronounced papulopustular eruptions resembling rosacea or acne, and sometimes blepharitis when involving the eyelids [2]. A related species, *Demodex brevis*, typically burrows into sebaceous glands, including the meibomian glands, leading to dry eye disease [1,5].

We report a rare case in which a heavy infestation of *D. folliculorum* was identified as the underlying cause of a chronic nipple sinus tract and associated nipple retraction in a young woman, initially misdiagnosed as an inflamed breast cyst.

## Case presentation

**Figure 1:**
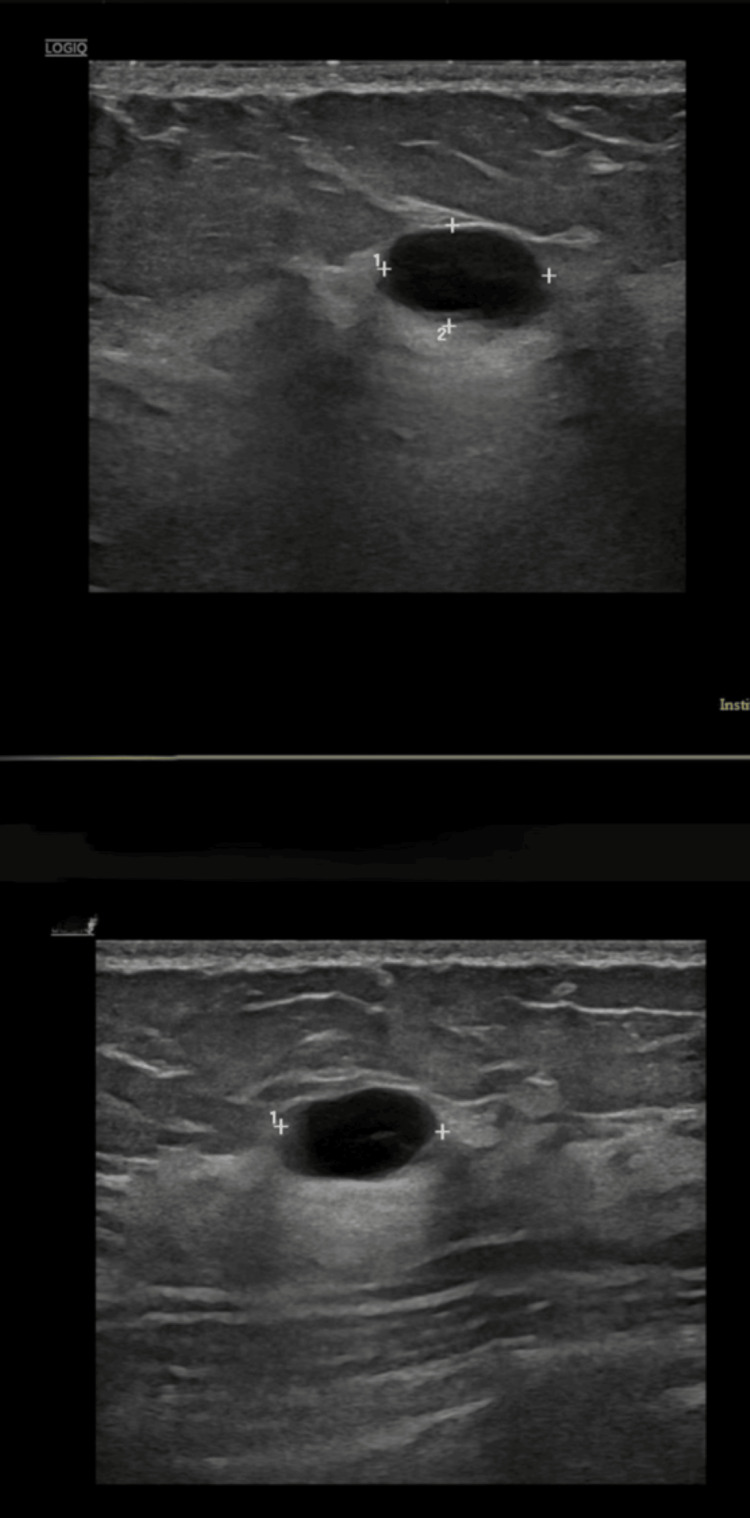
Breast ultrasound shows cystic lesions measuring 10 mm and 8.2 mm at the 2-3 o’clock position in the left breast (suggestive of simple cysts).

A 30-year-old, single, non-smoking, healthy Bahraini woman with no significant medical history, except that she had received three doses of the Pfizer COVID-19 vaccine in 2021, presented on February 17, 2022, with a three-day history of left breast pain and redness. Ultrasound revealed two adjacent superficial simple cystic lesions (10 mm and 8.2 mm) at the 2-3 o'clock position (Figure 1). Aspiration of the cysts yielded serosanguinous fluid. Fine-needle aspiration cytology (FNAC) was suggestive of an inflamed cyst. She was treated empirically with amoxicillin-clavulanic acid, and symptoms improved. Bacterial culture was negative at that time.

In June 2022, she experienced a severe COVID-19 infection. Four months later (in October 2022), her symptoms recurred, and she started complaining of painless, bloody discharge from a small ulcer in the left periareolar region. It was located at the site of the previous cyst aspiration. General examination revealed acne-like lesions on her forearms and back. Left breast examination showed a small periareolar skin ulcer at the 2 o’clock position, with surrounding redness approximately 1 cm in diameter and associated nipple retraction. No definite breast lump or axillary lymph nodes were palpable. A repeat breast ultrasound demonstrated a 1 × 0.9 cm collection confined within the skin of the left areola at the 1 o’clock position, with no deeper collection identified.

**Figure 2:**
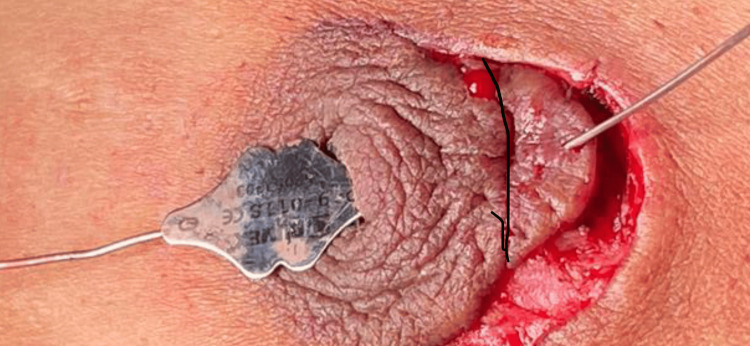
Intra-operative picture showing the probe passed through the retracted left nipple to the site of the inflamed cystic area (the black line was drawn to show the periareolar line).

The patient started complaining of itching and requested excision of the cyst along with correction of the retracted nipple. On October 31, 2022, the patient underwent surgical exploration. Intraoperatively, probing of the retracted nipple revealed a sinus tract extending easily from the nipple orifice to the periareolar ulcer at the 2 o’clock position (Figure 2). We excised the sinus tract, including the periareolar ulcer, raised the retracted nipple, and closed primarily. 

**Figure 3:**
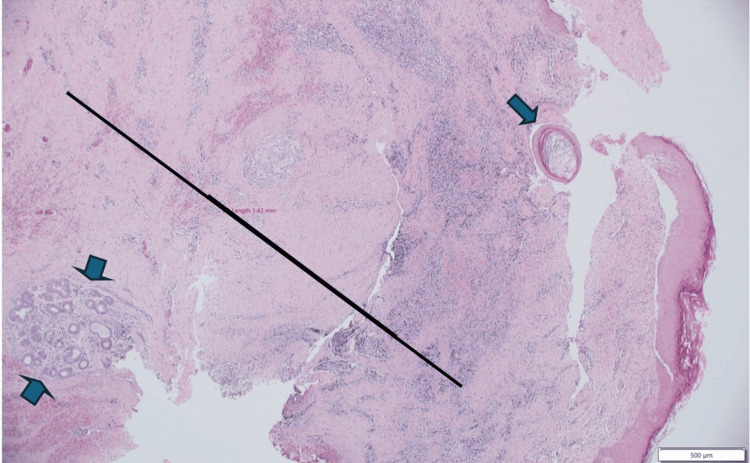
Excisional biopsy of the retro areolar skin lesion. The sinus tract is formed by an intact, tube-like superficial part lined by stratified squamous epithelium (arrow), and a deep, ruptured part formed by a chronic granulation tissue reaction (black line). Note the excess lymphoplasmacytic-rich inflammation, variable degrees of associated dermal scarring, and involvement of deep apocrine sweat glands (double arrows) (hematoxylin & eosin stain, low-power field).

**Figure 4:**
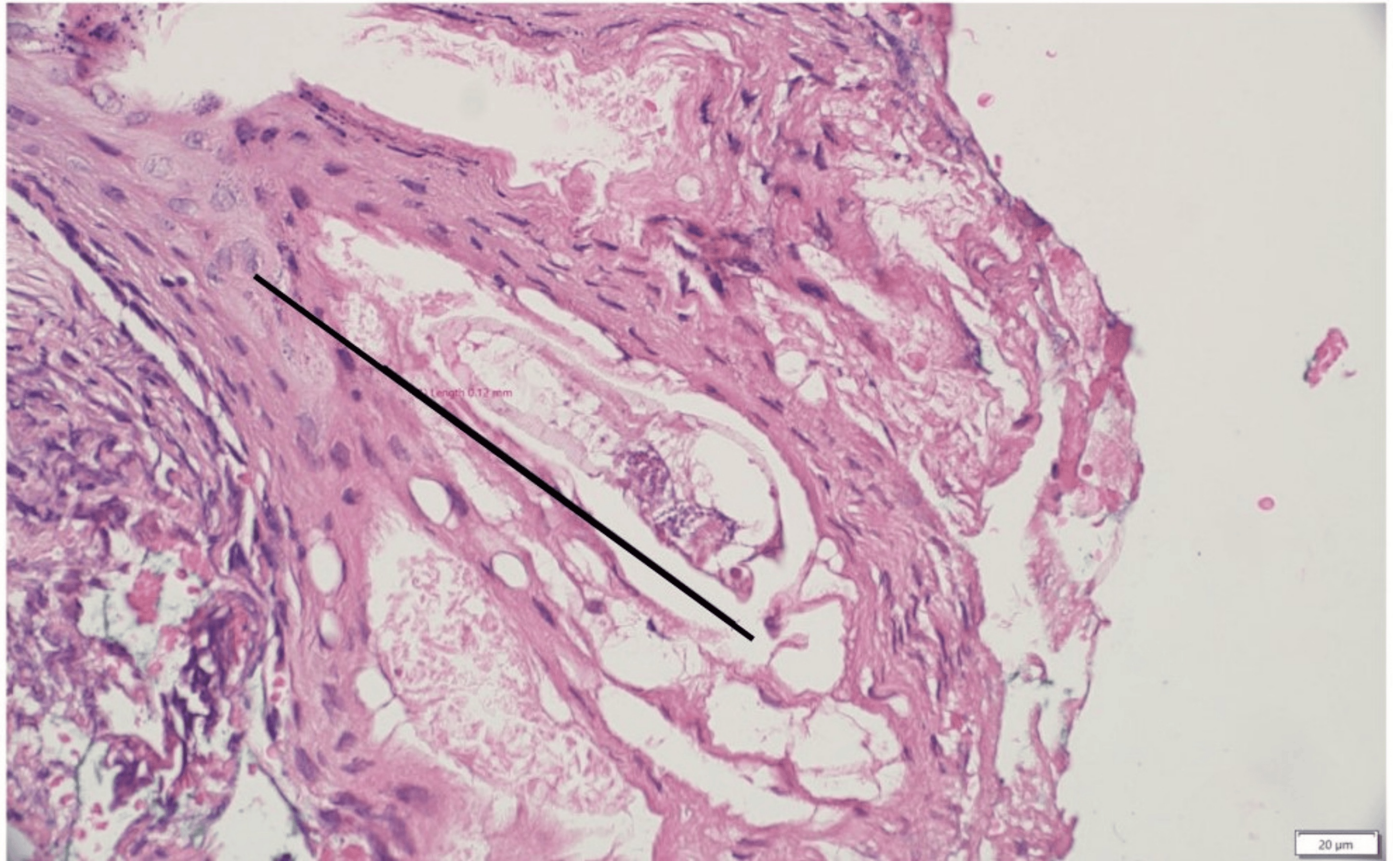
Excisional biopsy of the retro areolar skin lesion. Demonstrating the infested pilosebaceous acrosyringium by *Demodex folliculorum* mite: the hyperplastic epidermis harbors the ectoparasite, shown in a full-length cross-section (black line) (hematoxylin & eosin stain, medium-power field).

Histopathological examination of the excised sinus tract and ulcer tissue provided the definitive diagnosis. Microscopic sections showed a dense dermal, chronic granulation tissue reaction. The overlying epidermis was hyperplastic and contained milia-like microcysts with pilosebaceous units heavily infested by *D. folliculorum* mites. These findings were interpreted as a chronic nipple skin sinus associated with significant *Demodex* infestation (Figures 3-4).

**Figure 5:**
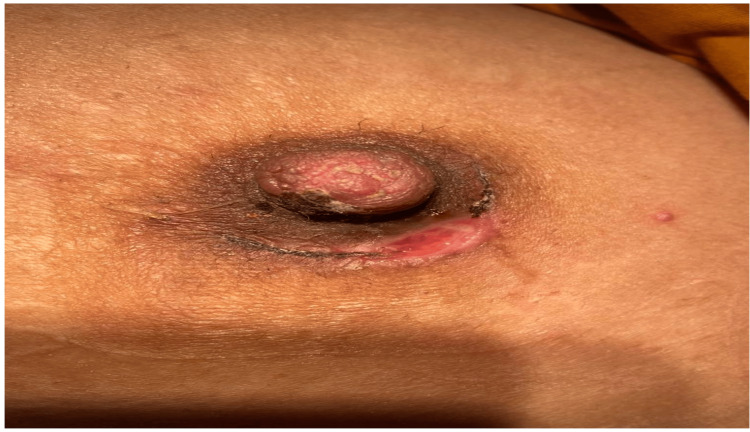
One week post-operative, the nipple was normal despite a superficial infection with skin gaping, which healed completely.

Postoperatively, the patient had superficial wound gaping which resolved with routine wound care (Figure 5).

On her follow-up visit in December 2022, the left breast wound had completely healed, and the nipple retraction had resolved. She was started on a two-week course of oral metronidazole, with topical use of tea tree oil body wash and sulfur soap. However, the itchy, acne-like lesions on her forearms and back persisted. A biopsy was recommended, but the patient declined further investigation.

## Discussion

*D. folliculorum* is an ectoparasite that inhabits human hair follicles and is more prevalent in males than in females. The pathophysiological mechanism of infection involves follicular distention and blockage by the mites, which feed on follicular cells [1]. Distention of follicular walls leads to antigen release. Epithelial hyperplasia and a host inflammatory response occur, potentially triggered by the mites themselves, their waste products, or associated bacterial infections such as *Staphylococcus* and *Bacillus oleronius*, which *D. folliculorum* mites carry [1]. Demodicosis often causes erythema, scaling, pruritus, papules, and vesicles within and around the infested follicles. This chronic inflammation involves both humoral and cell-mediated immune responses [2]. This can lead to tissue breakdown and the formation of sinus tracts. It seems likely that, under normal circumstances, there is a control mechanism that limits the population of *Demodex* mites. However, both local and systemic factors may create an environment that encourages their proliferation [6]. In general, infestation with *Demodex* mites is usually asymptomatic, but may be associated with skin disorders of the face, hyperpigmented patches, blepharitis, perioral dermatitis, or pustular folliculitis [6].

Large numbers of *Demodex* mites can be seen in the skin lesions; their response to topical therapy with permethrin indicates their pathogenicity. Breckenridge (1953) found *Demodex* mites in 13% of routine skin biopsies from all parts of the body [7]. He noted that these mites rarely caused chronic inflammation of the nipple-areolar complex. Early reports by Garven and Glasg (1946) highlighted the existence of these mites in the human nipple [8]. Subsequent studies, such as Val-Bernal et al. (2010), identified *Demodex* incidentally in nipple-areolar complex autopsies [9], and Kuleshova et al. (2017) detected mites in nipple secretions [10], confirming their presence in this atypical location.

Furthermore, Hoda and Cheng (2019) presented a case linking *Demodex* infestation to nipple pruritus, suggesting a potential role in localized symptoms [11]. Fidler (1978) found *Demodex* mites in 15 nipples [6]. They were seen in hair follicles or sebaceous ducts, but not in nipple ducts. *Demodex* mites play a significant role in the pathogenesis of eczema-like eruptions of the nipple [6]. Some patients with thick nipple discharge have shown *D. folliculorum* in nipple discharge cytology [6]. Demodicosis should also be included in the differential diagnosis of nipple eczema or nevoid hyperkeratosis [6,12].

Diagnosis of nipple demodicosis can be challenging due to its rarity and nonspecific presentation, particularly in a young, non-smoking, healthy woman. Histopathological examination of biopsied tissue is often required for confirmation, demonstrating mites within the follicular units and usually showing a perifollicular inflammatory reaction [2].

Our patient's initial presentation was consistent with an inflamed cyst, but the recurrence with skin ulceration and nipple retraction later necessitated further investigation. This nipple retraction likely resulted from chronic inflammation and fibrosis, associated with the sinus tract tethering the nipple inward.

The correct diagnosis in this case was only possible via histopathology after surgical exploration of the sinus tract. This highlights the importance of considering atypical etiologies and obtaining tissue biopsies for histology in chronic, non-healing, or recurrent nipple-areolar lesions. The differential diagnosis of chronic inflammatory or ulcerative lesions in the nipple-areola region should include other serious pathologies, such as Paget's disease of the nipple, ductal carcinoma in situ, nipple adenoma, and dermatological conditions like eczema or psoriasis [12].

The treatment approach worked very well in our patient by combining surgical excision of the affected tissue (sinus tract and ulcer) with anti-parasitic medication (oral metronidazole) and topical agents known to have activity against *Demodex* (tea tree oil and sulfur). Other reported treatments include topical 5% permethrin cream and hypochlorous acid (HOCl), with personal hygiene emphasized as crucial for management [1,2]. The successful resolution of the breast symptoms supports the diagnosis and the efficacy of the targeted treatment. Personal hygiene is considered one of the most important factors in eradicating these parasites [6].

To the best of our knowledge, chronic nipple-areola sinus secondary to demodicosis has never been reported in Bahrain, which posed a challenge to us in the differential diagnosis of this case. The patient’s history of a severe COVID-19 infection preceding the recurrence warrants mention, although a direct causal link cannot be established. Immune deficiency following COVID-19 infections has been postulated to exacerbate conditions like demodicosis [13]. The persistence of itchy lesions elsewhere on our patient’s body, which she declined to have biopsied, raises the possibility of a more generalized demodicosis.

## Conclusions

*D. folliculorum* infestation, though commonly reported on the skin, is a rare cause of chronic nipple and areolar pathology, particularly in our part of the world. This young, healthy woman presented with a chronic discharging sinus and associated nipple retraction following a COVID-19 infection, which likely lowered her immunity. Histopathological examination of excised tissue is essential for a definitive diagnosis. Treatment targeting the *Demodex* mites, along with surgical excision of chronically affected tissue, can lead to complete resolution of symptoms. Clinicians should include nipple-areola demodicosis as one of the differential diagnoses in such patients.
